# Circular RNAs in organ injury: recent development

**DOI:** 10.1186/s12967-022-03725-9

**Published:** 2022-11-18

**Authors:** Ryan Wong, Yiwen Zhang, Hailin Zhao, Daqing Ma

**Affiliations:** 1 Division of Anaesthetics, Pain Medicine and Intensive Care, Department of Surgery and Cancer, Faculty of Medicine, Imperial College London, Chelsea and Westminster Hospital, London, SW10 9NH UK; 2 Department of Anesthesiology, Shunde Hospital, Southern Medical University (The First People’s Hospital of Shunde, Foshan), Foshan, 528308 China

**Keywords:** circRNA, miRNA sponge, Gene expression regulation, Organ injury

## Abstract

Circular ribonucleic acids (circRNAs) are a class of long non-coding RNA that were once regarded as non-functional transcription byproducts. However, recent studies suggested that circRNAs may exhibit important regulatory roles in many critical biological pathways and disease pathologies. These studies have identified significantly differential expression profiles of circRNAs upon changes in physiological and pathological conditions of eukaryotic cells. Importantly, a substantial number of studies have suggested that circRNAs may play critical roles in organ injuries. This review aims to provide a summary of recent studies on circRNAs in organ injuries with respect to (1) changes in circRNAs expression patterns, (2) main mechanism axi(e)s, (3) therapeutic implications and (4) future study prospective. With the increasing attention to this research area and the advancement in high-throughput nucleic acid sequencing techniques, our knowledge of circRNAs may bring fruitful outcomes from basic and clinical research.

## Background

Circular ribonucleic acids (circRNAs) are endogenous single-stranded transcripts that are abundantly expressed in a cell-type-specific manner across all eukaryotic cells [1]. They lack a 5′ cap and a free-3′ tail and form a covalently enclosed loop. This molecular characteristic gives circRNAs a high resistance towards exonucleolytic degradation and relatively strong stability both intra- and intercellularly [2].

Initially detected in human HeLa cells in 1979 [3], circRNAs were once thought of as transcription byproducts. With the lack of high-throughput RNA sequencing and RNA genome mapping techniques, researchers were unable to fully comprehend the biological function of this unique RNA for the last 2 decades [4]. However, with the recent advancements in high-throughput techniques and computational bioinformatics analysis, researchers are now able to isolate, map and analyse circRNAs, revealing an exciting, unexplored field for research [5].

Recent studies suggested that circRNAs may play critical regulatory roles in various pathogenesis and disease pathways including cancer [6] and autoimmune diseases [7]. These include but are not limited to, regulating gene expressions, modulating protein–protein/RNA–protein interactions, and coordinating intracellular signalling pathways [2]. Importantly, recent studies also suggested that circRNAs may exhibit these regulatory roles in various organ diseases and injuries such as cardiovascular diseases [8], renal diseases [9], liver diseases [10], neurological diseases [11], and central nervous system (CNS) injuries [12]. These exciting findings highlight the potential roles of circRNAs in contributing to disease development and organ injury progressions. Hence, this review offers a summary of the current studies regarding circRNAs in organ injuries with respect to their therapeutic implications and future prospects.

### Biogenesis of circRNA

**Fig. 1:**
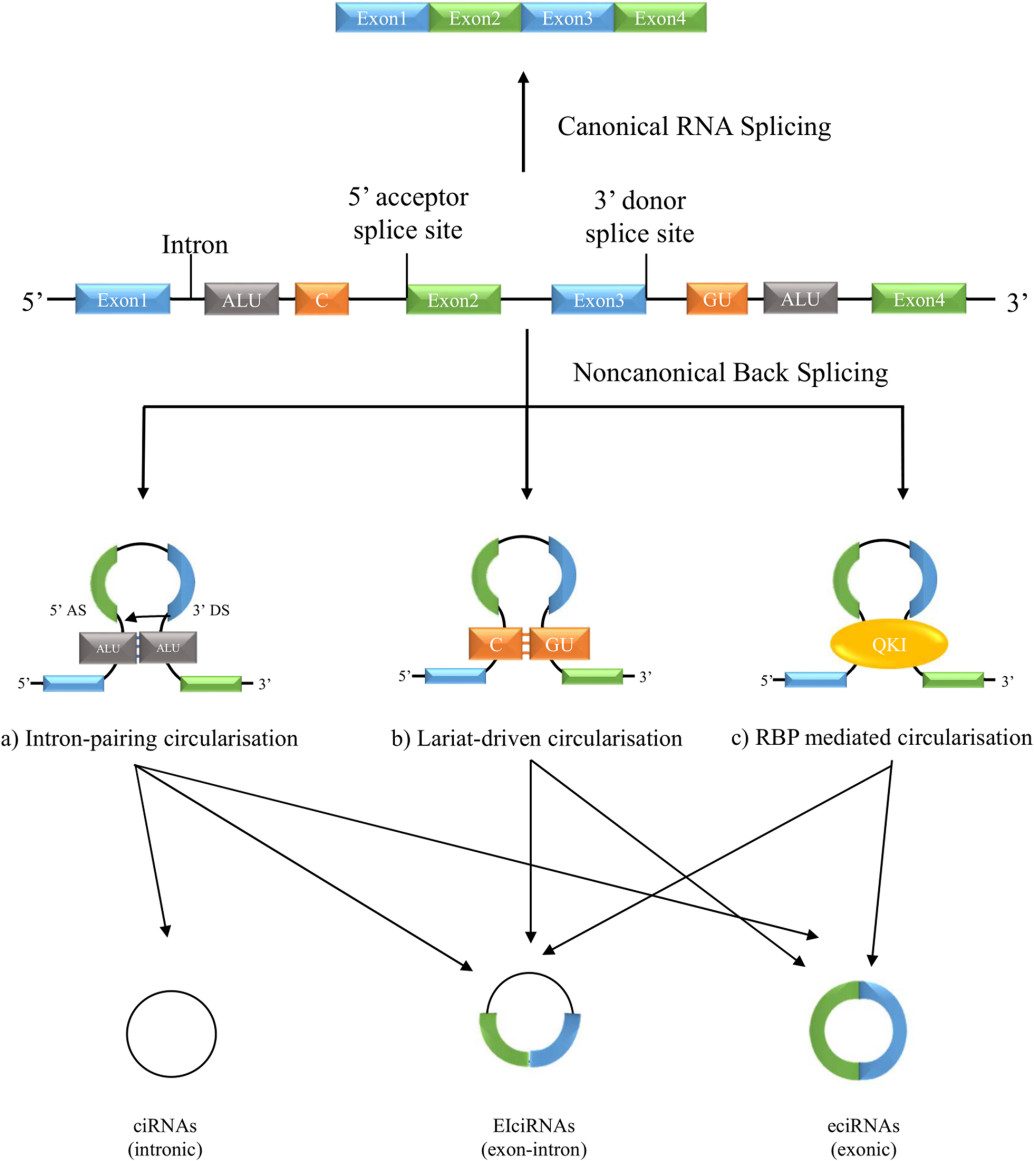
Schematic illustration of three biosynthesis pathways of circular RNA. Messenger RNA can be spliced through canonical splicing to produced mature mRNA and non-canonical splicing to produce circular RNAs. There are three back splicing models in non-canonical splicing that produced three types of circular RNAs. **a** The intron-pairing circularisation that produces all three types of circular RNAs: circular intronic RNAs (ciRNAs), exon–intron circular RNAs (EIciRNAs), and exonic circular RNAs (ecircRNAs). **b** The lariat driven circularisation that produces two types of circular RNAs: EIicRNAs, ecircRNAs. **c** The RNA binding protein mediated circularisation that produces two types of circular RNAs: EIicRNAs, ecircRNAs. Of which, ciRNAs are derived from introns and localise within the nucleus; EIciRNAs are derived from both exons and introns and localise within the nucleus; ecircRNAs are derived from exons and localise within the cytoplasm [150]

Different from canonical RNA splicing, circRNAs are generally synthesised through a back splicing mechanism. This involves the joining of a downstream 3′ donor splicing site with an upstream 5′ acceptor splicing site into a covalently enclosed single-stranded loop [13]. There are three current back splicing models which yield three types of nuclear genome-derived circRNAs, namely, circular intronic RNAs (ciRNAs), exon–intron circRNAs (EIciRNAs), and exonic circRNAs (ecircRNAs) [14] (Fig. 1). While there is also another type of circRNA—mitochondrial genome-derived circRNAs—its roles and functions in organ injuries were less studied. As a result, its biological and clinical characteristics remain largely unknown [15] and so will not be discussed in this review.

The first back splicing model—the intron-paired circularisation—is a direct back splicing mechanism. This is mediated by the flanking of intronic, reversed complementary sequences (e.g., ALU repeats). Upon splicing, the flanking reverse sequences from distinct intronic regions come in proximity and form complementary base pairings to bring the downstream donor splice site to the upstream acceptor site. The resulting RNA loop forms a secondary pre-mRNA structure that is readily available to be spliced at the joined exons point, resulting in the circularisation of RNA [16].

The second back splicing model—the lariat-driven circularisation—is an exon-skipping model. This model involves two steps of splicing events. The first event, similar to the intron self-splicing mechanism, is initiated by an adenosine branch point residue at a downstream intron site where the 2′-OH of the adenosine residue performed a nucleophilic attack to the GU region of an upstream intron. This joins the 3′ end of an upstream exon with a downstream exon, generating an intron lariat. The released intron lariat loop then further promotes RNA circularisation [17].

The last model is the RBP-mediated model. In this pathway, specific trans-acting RBPs—quaking (QKI)—bind to two distinct flanking intron regions and act as a bridge in between the 3′ donor splice site with the 5′ acceptor splice site to bring them into proximity. This promotes circularisation and thus results in the formation of a closed-loop RNA structure [18].

### Molecular mechanisms

**Fig. 2:**
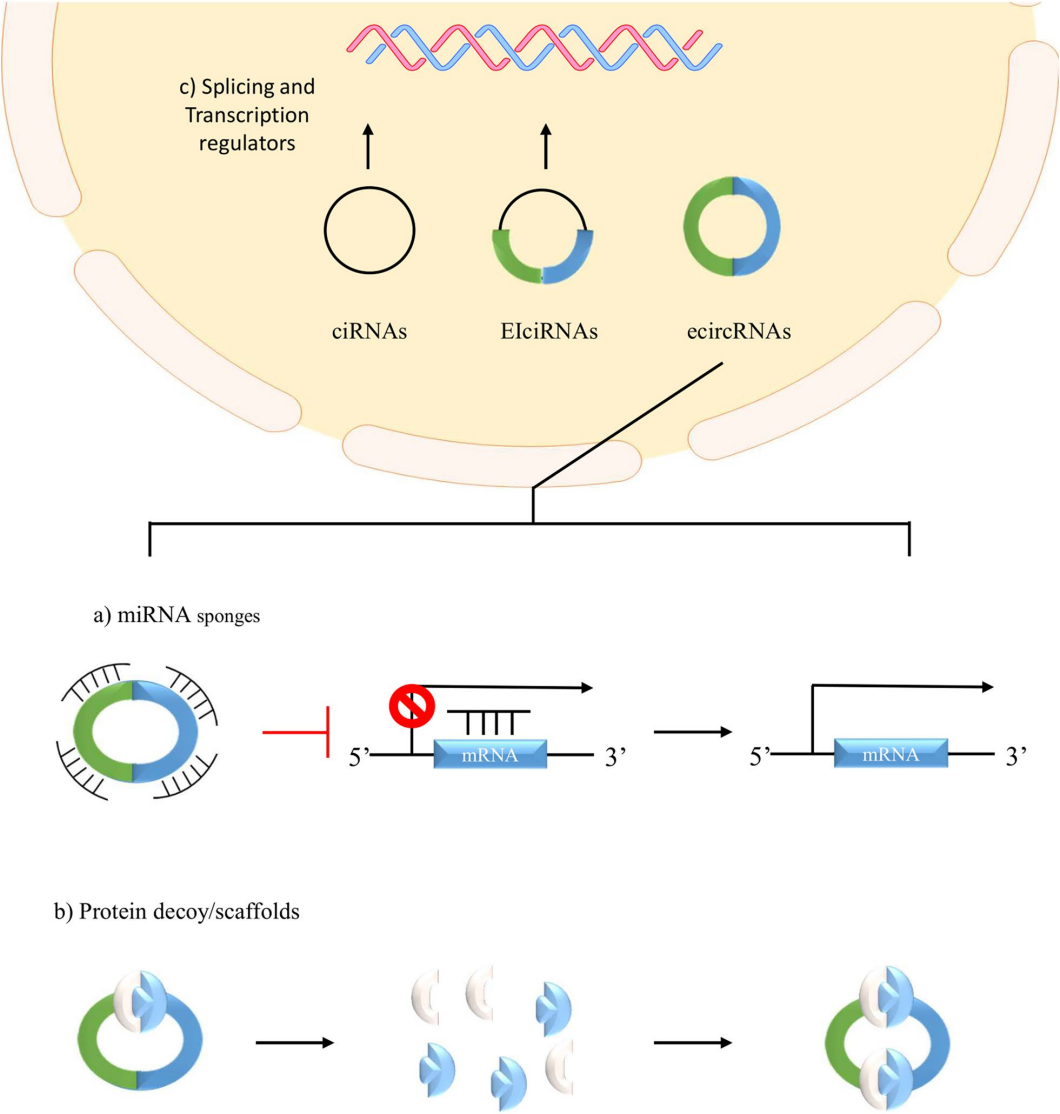
Schematic illustration of the molecular mechanisms of circular RNAs. Circular RNAs have been suggested to participate in regulating biologically important pathways. It does so through **a** sponging miRNA to regulate RNA expression; **b** acting as protein decoys and scaffolds to facilitate protein–protein/protein-RNA interactions; **c** acting as regulatory elements for transcription and mRNA splicing

Our current understanding of circRNAs suggested that they regulate biological pathways through two major mechanisms. Firstly, circRNAs can act as microRNA (miRNA) sponges to regulate target gene expressions. Secondly, circRNAs can act as protein decoys and scaffolds to modulate intracellular protein/protein and protein/RNA interactions [19] (Fig. 2). Although studies also suggested that circRNAs exhibited other molecular functions such as splicing and transcription regulators [20], these functions of circRNAs in the context of organ injury was less studied. Hence, this review will only focus on the two major mechanisms of circRNAs mentioned above.

#### MicroRNA sponge

MicroRNAs are short non-coding RNAs that regulate gene expressions through binding transiently with their complementary base pairing messenger RNAs (mRNAs), thereby silencing downstream translations and gene expressions [21]. Through computational bioinformatic analysis, studies have identified multiple miRNA response elements (MREs) on circRNAs [16]. These enriched MREs regions give circRNAs the ability to act as competing endogenous RNAs (ceRNAs). Through MREs, circRNAs can harbour and inhibit miRNAs by acting as “sponges” that bind to complementary base pairs corresponding to specific miRNAs. Hence, the upregulation of circRNAs will induce a high sponging effect on miRNAs, thereby downregulating miRNAs activity and indirectly upregulating downstream gene expressions, reversing the silencing property of miRNAs [10]. Likewise, the downregulation of circRNAs will result in an upregulation of miRNAs that subsequently inhibit the downstream target gene expressions. For example, circRNA cerebellar degeneration-related 1 antisense (CDR1as) contains over 70 miRNA-7 complementary binding sites to downregulate and inhibit miRNA-7 [1]. The downregulation of miR-7 subsequently leads to the activation of downstream insulin secretion and cell proliferation pathways [17]. In addition to their sponging activity, circRNAs can also improve single-stranded miRNA stability in the cytoplasm. Through complementary base pairing, circRNAs can form transient secondary RNA structures with miRNAs. Therefore, circRNAs not only negatively regulate miRNAs but also maintained the stability of intracellular miRNAs networks within the dynamic gene expression system in the cell [22].

#### Protein decoy and protein scaffolds

CircRNAs can bind to proteins and modulate protein–protein interactions by acting as protein decoys. They function as a scaffold to mediate the assembly of protein complexes. Through colocalising proteins with their corresponding downstream target substrates, circRNAs can increase the local concentration of the protein–protein interactions. In particular, circRNAs and elcircRNAs act as a tripod that provides a specific local scaffold for protein–RNA interactions during transcriptional regulation [10]. The scaffold network can also downregulate protein interactions by sequestering specific proteins from their native environments. For example, circFOXO3 binds and traps cell division protein kinase 2 (CDK2) and cyclin-dependent kinase inhibitor 1 (p21) within the cytoplasm. This formed a circFOXO3-CDK2-p21 complex structure which attenuates cell cycle progression [23].

### Biological pathways

With the two different upstream interactions, circRNAs can indirectly regulate downstream mediator proteins expression which serves to activate or attenuate important biological pathways. In particular, previous studies of circRNAs in organ injuries suggested that circRNAs regulate three main cellular functions: cell proliferation, apoptosis and inflammation [24].

#### Cell proliferation

CircRNAs may regulate cell proliferation through the canonical mitogen-activated protein kinases (MAPK) and Wnt/β-catenin pathway. The MAPK pathway regulates diverse cellular responses including cell proliferation. Upon activation, cell surface receptors such as receptor tyrosine kinases (RTK) are dimerised. This leads to autophosphorylation of the tyrosine residue in the intracellular regions of the receptors. This creates a phosphorylated docking site for adaptor proteins such as growth factor receptor-bound protein 2 (Grb2) to dock and recruit sone of sevenless (SOS). SOS, as a guanine nucleotide exchange factor (GEF), activates Ras. Activated Ras acts through the conventional ERK1/2 axis to phosphorylate ERK. Activated ERK can now translocate into the nucleus and regulate key cellular functions such as proliferation [25]. On the other hand, upon activation of the Wnt/β-catenin pathway, the Wnt ligands induce colocalisation of the two transmembrane receptors—Frizzled (FZD) and low-density lipoprotein receptor-related proteins 5 and 6 (LRP5/6)—and recruit a destruction complex composed of adenomatous polyposis coli (APC), AXIN, casein kinase 1 (CK1) and glycogen synthase kinase 3 (GSK3) protein. This recruitment attenuates the complex’s ability to degrade β-catenin. This allows intracellular β-catenin to translocate into the nucleus and activates the target gene via interaction with the T-cell factor (TCF) and lymphoid enhancer-binding factor (LEF), leading to the activation of the canonical pathway that promotes cell proliferation [26]. Recent studies have suggested that circRNAs may act on these two pathways via promoting or attenuating the expression of specific signalling proteins within the two axes. For example, circSnrk was suggested to act through the MAPK pathway to promote cell proliferation in alleviating AKI [27] while circRNA_Maml2 was demonstrated to upregulate the expression of FZD receptors to promote the activation of Wnt/β-catenin pathway during the repair of intestinal mucosa in intestinal injury [28].

#### Cell apoptosis

CircRNAs may also regulate cell apoptosis through the canonical phosphoinositide-3-kinase (PI3K)/protein kinase B (Akt) pathway. In the PI3K/Akt pathway, upon ligand-induced activation, the catalytic domain of PI3K catalyses the conversion of phosphatidylinositol [3, 4]-bisphosphate (PIP2) lipids to phosphatidylinositol (3,4,5)-trisphosphate (PIP3). PIP3 then binds to Akt to form a partially active activation loop to activate Akt. The activated Akt then phosphorylates Bcl-2-associated death promoter (BAD) and Bcl-2-associated X protein (Bax) to inhibit their pro-apoptotic effects and thus attenuate apoptosis [29]. An example of circRNAs acting on this axis can be seen from circ_0030235 in myocardial ischemia/reperfusion injury (I/RI) whereby it acts through the miR-526b axis to inhibit PI3K activity to promote apoptosis [30]. Similarly, studies have suggested that circRNAs may regulate this pathway through modulating the expression of B-cell lymphoma-2 (BCL2). For example, circUSP36 was suggested to act via the miR-139-3p/Smad3/BCL2 axis to positively regulate BCL2 and promote apoptosis [31].

#### Inflammatory responses

CircRNAs regulate inflammatory responses through the canonical nuclear factor kappa-light-chain-enhancer of activated B cells (NF-κB) pathway. The NF-κB pathway plays a pivotal role in mediating inflammatory response during both innate and adaptive immunity. Upon activation to pattern-recognition receptors (PPRs) or cytokine receptors, the intracellular domain of the receptors interacts with the inhibitor of nuclear factor kappa-B-kinase (IKK- B) complex. This subsequently leads to the phosphorylation, ubiquitination and degradation of IκBα, a catalytic subunit of the IKK complex. The remaining NF-κB dimer is then able to translocate into the nucleus, acting as a transcription factor to regulate target inflammatory genes expressions [32]. CircRNAs were demonstrated to regulate the NF-κB pathways. For example, circLRP6 was shown to act via the miR-205/high mobility group box 1 (HMGB1) axis to regulate the activation of NF-κB pathway in mesangial cell injury. This serves to promote cell proliferation and alleviate mesangial cell injury in kidney [33].

### CircRNAs in organ injury

**Fig. 3:**
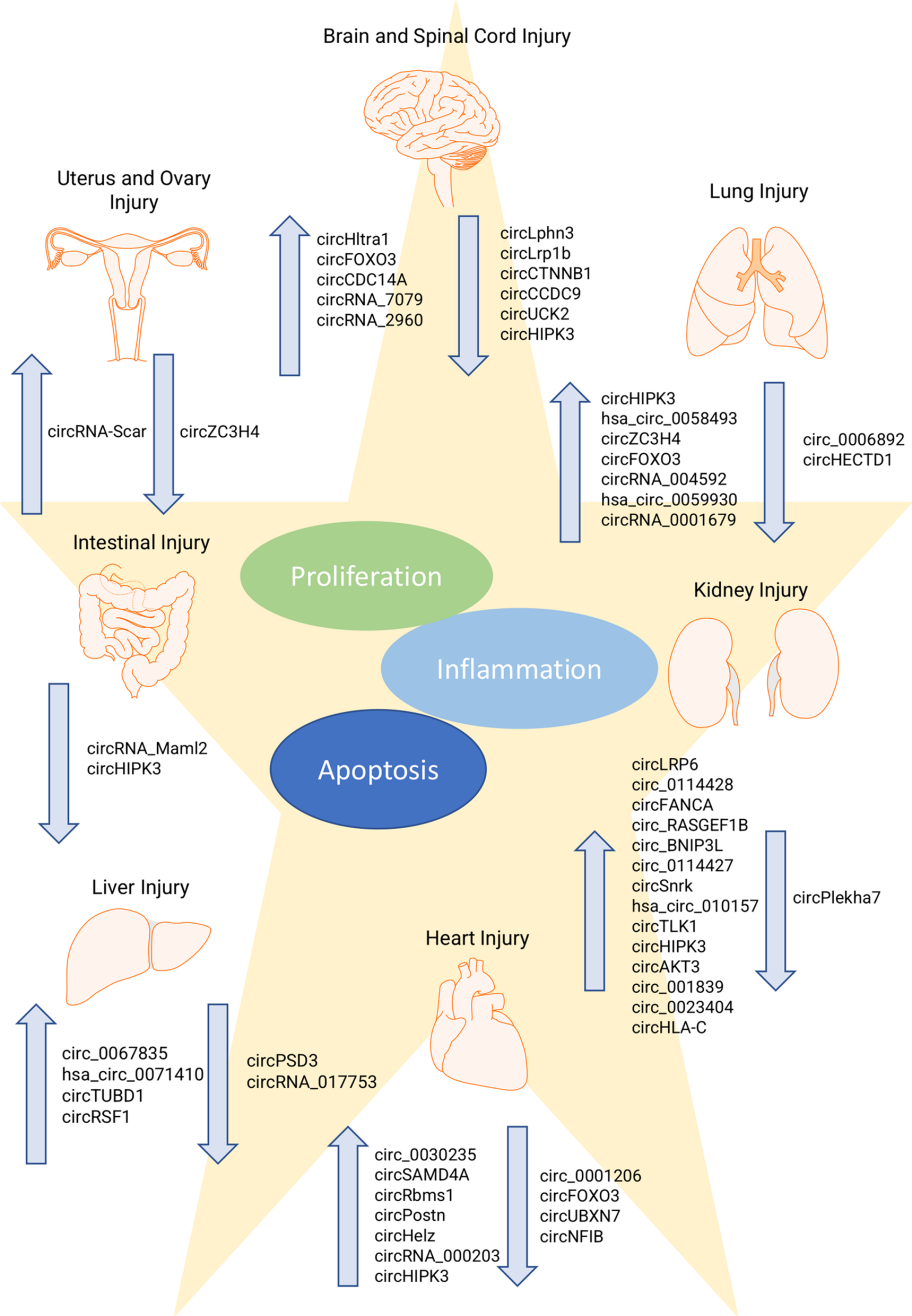
Schematic illustration of circular RNAs expression pattern across major types of organ injuries. The figure summarises all the recent reported dysregulated circular RNAs across each type of organ injury. Together, the up- and downregulated circular RNAs contributed to three main biological functions: cell proliferation, cell apoptosis, and inflammation. These pathways may result in either alleviating or aggravating organ injuries. Each of the circular RNA and its respective roles are discussed in the paper

At a cellular scope, organ injuries involve various types of pathological processes: from cell death to inflammatory responses towards physiological repairing cell proliferation or pathological fibrogenesis. CircRNAs may play important regulator functions in each of these stages and are associated with both disease aggravation and alleviation [24]. The following sections aim to present a summary of key circRNAs studied in each organ injury and their roles respectively (Fig. 3; Table 1).

**Table 1:** Summary of dysregulated circular RNAs in organ injuries

Organ	Types of injury	CircRNA expression pattern	Downstream regulation	References
*CNS*				
Brain	Traumatic brain injury	circLphn3 downregulated	Improved BBB integrity and promoted inflammatory response	[43]
		circLrp1b downregulated	Inhibits inflammatory response	[45]
		circHtra1 upregulated	Inhibits cell proliferation and promotes cell apoptosis	[46]
	Ischemia/reperfusion injury	circCTNNB1 downregulated	Improves BBB integrity and promotes cell proliferation	[50]
		circFOXO3 upregulated	Promotes autophagy and maintains BBB integrity	[51]
		circCCDC9 downregulated	Improves BBB integrity	[52]
		circUCK2 downregulated	Inhibits cell proliferation and promotes cell apoptosis	[53]
		circCDC14A upregulated	Promotes cell proliferation and migration	[56]
		circUSP36 downregulated	Inhibits cell apoptosis and promoted cell proliferation	[31]
Spinal cord	Spinal cord injury	circHIPK3 downregulated	Inhibits cell proliferation and promotes cell apoptosis	[60]
		circRNA_7079 upregulated	Promotes inflammatory response	[63]
		circRNA_2960 upregulated	Promotes inflammatory response and inhibits cell apoptosis	[65]
*Visceral organ*				
Lung	Idiopathic pulomnary fibrosis	circHIPK3 upregulated	Promotes fibroblast proliferation and lung fibroblast-to-myofibroblast transition	[68]
		hsa_circ_0058493 upregulated	Potential biomarker candiate	[69]
	Silicosis	circZC3H4 upregulated	Regulates fibroblast activation and proliferation	[71]
	Cigarette particle-induced lung injury	circFOXO3 upregulated	Promotes inflammatory response	[74]
		circRNA_0006892 downregulated	Promotes cell apoptosis and inflammatory response	[75]
	Pulmonary hypertension	circRNA_004592 upregulated	Promotes cell proliferation and inhibits cell apoptosis	[79]
	Acute lung injury	hsa_circ_0059930 upregulated	Inhibits cell apoptosis and inhibits cell proliferation	[81]
		circRNA_0001679 upregulated	Promotes cell apoptosis and proinflammatory cytokines	[82]
		circHECTD1 downregulated	Inhibits cell apoptosis	[83]
Kidney	Mesangial cell Injury	circLRP6 upregulated	Promotes inflammatory response	[33]
	Acute kidney injury	circ_0114428 upregulated	Promotes inflammatory response	[90]
		circFANCA upregulated	Promotes cell apoptosis, inflammatory response, oxidative stress	[91]
		circ_RASGEF1B upregulated	Promotes cell apoptosis and inflammatory response	[92]
		circ_BNIP3L upregulated	Promotes inflammatory response and oxidative stress	[93]
		circ_0114427 upregulated	Promotes inflammatory response	[94]
		circSnrk upregulated	Promotes cell apoptosis	[27]
		hsa_circ_010157 upregulated	Promotes cell apoptosis and inflammatory response	[95]
		circTLK1 upregulated	Promotes cell apoptosis, inflammatory response, oxidative stress	[96]
		circHIPK3 upregulated	Promotes cell apoptosis and inflammatory response	[97]
	Ischemia/reperfusion kidney injury	circAKT3 upregulated	Promotes cell apoptosis	[99]
		circ_001839 upregulated	Promotes inflammatory response	[100]
		circ_0023404 upregulated	Promotes cell apoptosis and inflammatory response	[101]
	Renal fibrosis	circPlekha7 downregulated	Promotes cell proliferation	[104]
	Lupus nephritis	circHLA-C upregulated	Promotes lupus nephritis pathogenesis	[106]
Heart	Myocardial ischemia–reperfusion injury	circ_0030235 upregulated	Promotes cell apoptosis	[30]
		circ_0001206 downregulated	Promotes cell apoptosis	[109]
		circSAMD4A upregulated	Promotes cell apoptosis and inflammatory response	[110]
		circFOXO3 downregulated	Inhibits inflammatory response	[111]
		circRbms1 upregulated	Promotes FOXO1 expression	[112]
		circUBXN7 downregulated	Inhibits cell apoptosis and inflammatory response	[113]
		circPostn upregulated	Promotes cell apoptosis	[115]
		circHelz upregulated	Promotes inflammatory response	[116]
	Cardiac fibrosis	circNFIB downregulated	Promotes fibroblast proliferation and cardiac fibroblast-to-myofibroblasts differentiation	[118]
		circRNA_000203 upregulated	Promotes α-SMA, Col1a2 and CTGF expression	[120]
		circHIPK3 upregulated	Promotes α-SMA, Col1a1 and Col3a1 expression	[121]
Liver	Hepatic fibrosis	circPSD3 downregulated	Promotes inflammatory response	[123]
		circ_0067835 upregulated	Promotes cell proliferation	[125]
	Hepatic ischemia–reperfusion injury	circRNA_017753 downregulated	Activates Jade1 signalling pathway	[129]
	Radiation-induced liver Injury	hsa_circ_0071410 upregulated	Promotes cell proliferation	[131]
		circTUBD1 upregulated	Promotes inflammatory response	[133]
		circRSF1 upregulated	Promotes cell proliferation and inflammatory response	[136]
Intestine	Intestinal injury	circRNA_Maml2 downregulated	Promotes cell proliferation, differentiation and migration	[28]
		circHIPK3 downregulated	Inhibits cell proliferation	[139]
*Reproductive system*				
Uterus and ovary	Uterus and ovary injury	circZC3H4 downregulated	Promotes cell apoptosis	[141]
		circScar upregulated	Inhibits cell proliferation	[141]

The table summarised all the discussed circular RNAs in this paper. The expression patterns of each circular RNAs and their corresponding pathological outcomes in each organ injury were shown. Importantly, circFOXO3, circHIPK3 and circZC3H4 were consistently reported in more than one types of organ injuries

*Circular RNA*, circRNA; *CNS*, Central nervous system

## Central nervous system

Using next-generation sequencing, recent studies have identified a large pool of circRNAs that were involved in regulating cell inflammation, apoptosis, neuronal development, and autophagy in the CNS during post-traumatic injuries [34–38]. In particular, these circRNAs were mostly identified from traumatic brain injury (TBI) and spinal cord injury (SCI) [39].

### Brain

#### Traumatic brain injury

Traumatic brain injury (TBI) as a result of sudden trauma to the brain is one of the leading causes of disability worldwide [40]. It can occur in all age groups and lead to disability and even death. Interestingly, recent studies on circRNAs in TBI suggested that circRNAs may play an important role throughout the course of TBI including initial cell death, autophagy and inflammatory response to subsequent repairing process. Trauma to the brain results in dysfunction of the blood–brain barrier (BBB) integrity and activates inflammatory response following TBI [41]. A recent study using TBI mouse models identified a significantly downregulated circRNA—circLphn3—to play a role in regulating BBB integrity during TBI. Using dual-luciferase receptor assay, Liao et al. showed that circLphn3 acted via the miR-185-5p/zona occludens-1 (ZO-1) axis to modulate the expression of tight junction protein ZO-1. As a result, downregulation of circLphn3 negatively regulated the expression of ZO-1 and hence, improved BBB permeability. The increased BBB permeability as a result of trauma subsequently activated the cells to a pro-inflammatory state that promoted downstream inflammatory response in TBI [42]. In this study, the identified circLphn3 could be a potential early biomarker as the changes in its expressions occurred during the initial course of TBI. However, the study also highlighted they were only able to identify one interaction of circLphn3 through the miR-185-5p/ZO-1 axis. The study suggested that circlphn3 could potentially target alternative miRNAs to regulate other essential tight junction proteins such as ZO-2 and occluding. Hence, further studies are required to establish a clearer relationship between the potential players within the network and their respective regulatory functions before testing the therapeutic possibility of circLphn3 [43].

Furthermore, the roles of circRNAs in inflammation and autophagy were shown in another animal study. Using rat TBI models, Xie et al. reported four circRNAs to be significantly dysregulated in the mouse hippocampus upon piston-induced-TBI [44]. Further analysis in follow-up study showed that the dysregulated circRNA—circLrp1b—played a critical role in TBI-induced autophagy and inflammatory response. Through northern blot and luciferase reporter assays, the study showed that circLrp1b acted via the miR-27a-3p/Dram2 axis to positively regulate the expression of DNA damage regulated autophagy modulator 2 (Dram2) to promote autophagy activity. Taking the result further, the study co-administrated Dexmedetomidine (DEX) with circLrp1b and showed that DEX inhibited inflammatory response and autophagy by inactivating the circLrp1b/miR-27a-3p/Dram2 signalling pathway [45]. Together, these studies successfully demonstrated circLrp1b’s potential role as a therapeutic target and that attenuation of the circLrp1b/miR-27a-3p/Dram2 axis with DEX can reversed the overexpression of Dram2 and hence the autophagy and inflammatory response during TBI. However, the current study only offered limited prospective by studying one specific downstream axis of circLrp1b. Further studies are recommended to verify circLrp1b’s interaction in vivo and to create a more defined interaction network of circLrp1b with its downstream effector proteins. Moreover, in order to verify the whether the identified circLrp1b was TBI specific, additional studies should compare and contrast circRNA expression profiles in similar injuries or disease pathologies to confirm circLrp1b’s specificity as a biomarker for TBI.

In addition, the role of circRNA in regulating cell death was demonstrated by Zheng et al. Through analyses of the blood sample from TBI patients, circHtra1 was identified to be significantly upregulated. Further Gene ontology (GO) and Kyoto Encyclopedia of Genes and Genomes (KEGG) pathway analyses showed that circHtra1 acted via the miR-3960/GRB10 axis to positively regulate GRB10. As a result, GRB10 promoted apoptosis through regulating the canonical Wnt and β-catenin pathways [46]. Although the study successfully demonstrated the correlation of the circHtra1/miR-3960/GRB10 axis with inhibition of cell proliferation and the promotion of cell apoptosis, it failed to provide detailed molecular mechanisms of circHtra1’s role in this network. Further studies are recommended to further explored this function of circRNA in TBI.

Lastly, studies have also suggested that circRNAs play a role in the cellular repair process post-TBI. Using microarray analysis, Xie et al. generated a circRNA expression profile of TBI-induced mouse hippocampus. Further GO and KEGG pathway analyses suggested that circRNAs may play a role in regulating the regeneration of neurons, formation of glutamatergic synapses and guidance of axon growth post-TBI [47]. Interestingly, another study by Zhao et al. identified similar GO analysis matches from circRNAs derived from the extracellular exosomes of TBI-induced mouse brain. The study suggested that the extracellular circRNAs in brain-derived exosomes could involve in dendrite development and glutamatergic synapse formation [48]. Together, these two studies identified potential circRNA targets that are worth studying with respect to their functions as potential biomarkers.

#### Brain ischemia/reperfusion injury (I/RI)

Brain ischemia/reperfusion injury is a common result of ischemic stroke when the blood supply to the brain was interrupted for a period of time before restoration [49]. As a result of oxygen deprivation, brain I/RI could lead to different extents of severity depending on the deprivation time. Hence a potential biomarker to reflect the severity of brain I/R injury or a therapeutic target to reverse or attenuate the injury progression would greatly improve the prognosis for brain I/RI.

Similar to TBI, recent studies also suggested that circRNAs may play a role in regulating BBB integrity and cell proliferation in I/RI during ischemic stroke injury. Using in vitro oxygen–glucose deprivation and reperfusion (OGD/R) mouse astrocytes and in vivo mouse middle cerebral artery occlusion models, Chen et al. identified circCTNNB1 to be significantly downregulated during I/RI. Through overexpression of circCTNNB1, the study reversed the inhibition of scavenger receptor class B type 1 (SRB1) protein through the circCTNNB1/miR-96-5p/SRB1 axis [50]. While this study showed that overexpression of circCTNNB1 were able to alleviate I/RI, the molecular mechanisms behind SRB1’s functions remain unclear. As suggested in the study, further studies are recommended to investigate SRB1’s role in regulating oxidative stress and inflammation in I/RI.

Moreover, circRNA’s role in maintaining BBB integrity in brain I/RI is further evidenced by Yang et al. Using both ischemic stroke patient’s blood samples and mice models, the study identified an upregulation of circFOXO3 expression level during brain I/RI. Further analyses of circFOXO3 showed that by interacting with mTOR and E2F1, circFOXO3 was able to inhibit mTORC1 activity. This in turn promoted autophagy activity under I/RI and helped maintain BBB integrity, offering a protective function during I/RI [51]. Although this study successfully explored circFOXO3’s role as protein decoys, its role as miRNA sponges in brain I/RI remains unclear. Hence, further studies are needed to further verify circFOXO3’s potential interactions with miRNAs in brain I/RI.

In addition, study using the Transient middle cerebral artery occlusion (tMCAO) mice model also identified another circRNA—circCCDC9—to exhibit a similar function during brain I/RI. Through reverse transcription-quantitative polymerase chain reaction (rt-qPCR) and BBB integrity assays, Wu et al. identified circCCDC9 to be significantly downregulated during I/RI. Further analyses showed that circCCDC9 inhibited the expression of Caspase-3, Bax/BCL2 ratio and Notch1 to enhanced BBB integrity [52]. What’s more, a recent study suggested that circRNA may play a role in regulating apoptosis during cerebral I/RI. Using an oxygen-glucose deprived immortalised mouse hippocampal (HT22) cell model, Chen et al. identified circUCK2 to be significantly downregulated after cerebral I/RI. Luciferase reporter and RNA pull-down assay suggested that circUCK2 acted via the miR-125b-5p/growth differentiation factor 11 (GDF11) axis to positively regulate the expression of GDF11 [53]. As a member of the transforming growth factor-beta (TGF-β) superfamily, GDF11 is capable of activating the Smad3 signalling pathway [54]. The downstream effector, Smad3, was previously reported to have an anti-apoptotic effect in cerebral I/RI [55]. Interestingly, circUCK2 was reported to improve cell survival in cerebral I/RI through acting via the miR-125b-5p/GDF11 axis to inhibit apoptosis. While this study provided an insight to circUCK2’s critical role in cerebral I/RI, it also highlighted that miR-125b-5p has multiple downstream targets other than GDF11 such as sirtuin-1. To further study the therapeutic prospect of circUCK2, future studies are required to investigate into alternative downstream targets of circUCK2 to construct a clearer interaction network.

Apart from regulating cell apoptosis, studies also showed that circRNAs may play a role in promoting cell proliferation in cerebral I/RI. Huo et al. identified circCDC14A as significantly upregulated during OGD/R induced I/RI condition in HT22 cells. Through sponging miR-23a-3p, circCDC14A can positively regulate chemokine (C-X-C motif) ligand 12 (CXCL12) expression level to promote cell proliferation and migration [56]. Further study by Yang et al. also showed that an alternative circRNA—circUSP36—may also promote cell proliferation in ischemic stroke via a different axis. Using ischemic stroke patients’ peripheral blood samples, the study identified a significant decrease in the circUSP36 expression level. Further analyses showed that circUSP36 acted via the miR-139-3p/Smad3/BCL2 axis [31]. As a result, downregulation of circUSP36 attenuated the Smad3/BCL2 pathway which subsequently inhibited cell apoptosis and promoted cell proliferation, thereby attenuating ischemic stroke injury. Interestingly, the identified downstream effector of circUSP36, Smad3, is also a downstream effector of circUCK2. This may suggest that multiple circRNAs may exhibit similar functions during I/RI. However, further studies are required to validate this speculation. Further studies are also recommended to study circRNAs that act through similar axis together to better define our understanding of the interaction network of circRNA during I/RI in ischemic stroke.

#### Spinal cord injury

Spinal cord injury, as a result of sudden and traumatic damage to the spinal cord, can be an irreversible trauma with poor prognosis [57].

There are currently two main types of studies that looked at circRNAs in SCI: short recovery time and long recovery time after SCI. In short recovery time after SCI, Zhou et al. identified 150 circRNAs to be significantly differentially expressed in 6 h post SCI rat models. Further analyses identified 99 circRNAs as significantly upregulated and 51 circRNAs as significantly downregulated [58]. Through GO and KEGG pathway analyses, the upregulated circRNAs were found to reduce the pro-neuroinflammatory cascade in SCI by activating the peroxisome proliferator-activated receptor signalling pathway. Whereas the downregulated circRNAs were shown to modulate protein kinase binding, extracellular matrix-receptor interaction and synaptic vesicle exocytosis. The identified circRNAs in study provided guidance for further studies in investigating the underlying mechanism and interaction network of circRNAs in SCI.

In addition, a recent study by Liu et al. also reported that 1,998 circRNAs were significantly dysregulated with 1,101 circRNAs upregulated and 897 circRNAs downregulated during short recovery time after SCI [59]. In particular, one of the significantly downregulated circRNAs—circHIPK3—were further studied by Ye et al. through in vitro neuronal cell models. Using rt-qPCR, the study suggested that downregulation of circHIPK3 inhibited dual specificity phosphatase 1 (DUSP1) expression, thereby promoting inflammatory response [60]. Hence, overexpression of circHIPK3 could reverse DUSP1 inhibition and in turn attenuates inflammatory response and cell apoptosis to alleviate SCI. Although this study successfully identified a potential therapeutic target, the molecular interactions of circHIPK3 in SCI needs to be consolidated with in vivo models.

In long term recovery time after SCI, studies suggested that circRNA may play a role in regulating apoptosis and inflammatory response in SCI. Through contusion injury-induced SCI rat models, Qin et al. identified 415 significantly upregulated circRNAs and 1261 significantly downregulated circRNAs post-SCI [61]. Further GO and KEGG pathway analyses suggested that these circRNAs were related to the adenosine 5’ monophosphate-activated protein kinase (AMPK) signalling pathway. As previous studies reported, AMPK pathway was involved in the regeneration and proliferation of sensory neurons post SCI [62]. In addition, studies have also suggested that circRNAs may play a role in post-SCI neuroinflammation and apoptosis during long term recovery time after SCI. Yao et al. identified 909 circRNAs to be significantly upregulated in SCI mouse models [63]. In particular, downstream analyses of circRNA_7079 showed that it can positively regulate galectin 3 (Lgals3) through sponging mmu-miR-6953-5p. As a result, Lgals3 activated downstream reactive oxygen species (ROS)/thioredoxin-interacting protein (TXNIP)/NOD-, LRR- and pyrin domain-containing protein 3 (NLRP3) signalling pathway and thereby enhanced neuroinflammation [64].

Similarly, the same inflammatory function of circRNAs in long term recovery time post SCI was also reported by Chen et al. The study identified circRNA_2960 to be significantly upregulated in 7 days post-SCI mouse models. GO and KEGG pathway analyses suggested that this novel circRNA may promoted cell apoptosis and inflammatory response through sponging miRNA-124 [65]. However, the study was not able to identify the precise molecular mechanisms and the defined the downstream interactions. Further studies are recommended to investigate in the downstream effectors of miRNA-124 and its respective cellular functions.

Overall, current studies have successfully identified circRNAs’ roles in major CNS injuries such as TBI and SCI. Most studies suggested that circRNAs play a regulatory role in cell proliferation, apoptosis, inflammatory response and maintaining BBB integrity. For TBI, current studies had identified several circRNAs with potential therapeutic prospects. Importantly, many of the identified circRNAs shared similar regulatory functions and acted through similar axis such as circUCK2 and circUSP36. Hence, it is important to establish a clearer understanding in the regulation network of circRNAs in TBI as a whole in order to verify the therapeutic potential of circRNAs in TBI. For SCI, majority of the current studies on circRNAs remained preliminary. Further in vivo studies are needed to verify circRNAs’ roles in SCI.

### Lung

#### Idiopathic pulmonary fibrosis

Idiopathic pulmonary fibrosis (IPF) as a result of inhaling metal or wood dust or specific viral infections is a chronic, progressive disease that severally damages the lungs [66]. It has a poor prognosis and lacks effective treatments. Excitingly, recent studies have shown that circRNAs exhibit regulatory roles in pulmonary fibrogenesis and fibroblast activation, suggesting their therapeutic potential [67].

Zhang et al. identified significant upregulation of circHIPK3 expression in bleomycin-induced pulmonary fibrosis mice model. Luciferase receptor assay suggested that circHIPK3 may act via the miR-338-3p/SOXO4 and miR-338-3p/COL1A1 axes to promote fibroblast proliferation and lung fibroblast-to-myofibroblast transition, enhancing fibrogenesis in IPF [68]. Interestingly, the identified circRNA, as previously discussed, was also reported in the SCI study. However, circHIPK3 was reported to be upregulated in SCI while downregulated in pulmonary fibrosis. These differences in circHIPK3 expression levels and its respective signalling axes in different injuries may offer insights into the fundamental molecular mechanisms of circRNAs. Future studies should explore circHIPK3’s roles to a greater extent to help us understand whether circRNAs act in a tissue- and cell-specific manner or a function-specific manner.

Moreover, apart from circRNAs’ biological functions in IPF, a recent study also suggested that peripheral blood circRNAs could act as potential biomarkers for IPF and silicosis. Using RNA-sequencing technique, Cheng et al. reported that hsa_circ_0058493 was significantly upregulated in both silicosis and IPF peripheral blood samples [69]. Although this study revealed a potential circRNA as a biomarker, the sample size used in this study was too small: with only 3 IPF cases and 4 controls. Future studies should verify the translational value of hsa_circ_0058493 with a larger sample size.

#### Silicosis

Silicosis as a result of prolonged inhalation of crystalline silica dust is a fatal condition that leads to chronic lung inflammation and fibrosis [70]. Studies suggested that circRNAs may play a significant role during the inflammatory response in silicosis.

Through analyses of RAW264.7 macrophages and the alveolar macrophage samples from healthy donors and patients, circZC3H4, a circular derivative of the zinc finger CCCH-type containing 4 (ZC3H4), was found to be significantly upregulated in SiO2-activated macrophages [71]. As the name suggested, circZC3H4 acted via the ZC3H4 pathway to regulate fibroblast activation and proliferation [72]. However, the precise mechanism underlying this pathway is unclear and its potential therapeutic prospect is yet to be explored.

#### Cigarette particle-induced lung injury

Cigarette particle-induced lung injury accounts for over 85% of chronic obstructive pulmonary disease (COPD) and other non-neoplastic lung injuries including bronchiolitis/pneumonitis, bronchiectasis and emphysema [73]. Zhou et al. has identified a significant upregulation in circFOXO3 expression in cigarette particle-induced lung injury mouse models. Further analyses showed that circFOXO3 acted via the miR-214-3p/IKK-β axis to positively regulate IKK-β. Upregulation of IKK-β promoted the expression of cigarette particle extract-induced inflammatory cytokines such as CXCL1 and interleukin-6 (IL-6). Through knockdown assay, the study confirmed that circFOXO3 was a key regulator in pneumonic inflammation response during cigarette particle-induced lung injury [74]. In addition, another study by Zhang et al. also identified circRNAs to participate in the inflammatory response during cigarette particle-induced lung injury. The study reported a significant downregulation of circ_0006892 expression in cigarette particle extract-induced bronchial epithelial injury patient tissues. Downregulation of circ_0006892 reversed its sponging activity to miR-24 and resulted in the inhibition of PHLPP2. This subsequently promoted cell apoptosis and inflammatory response in cigarette particle-induced lung injury [75].

Together, these studies highlighted the underlying roles circRNAs play in the regulation of inflammatory response during cigarette particle-induced lung injury. However, as current studies were limited in mouse models only, further preclinical models are recommended to more accurately mimic and reflect on the microenvironment of the cigarette particle-induced lung injury in humans. This would help to verify the therapeutic prospect of circRNAs in cigarette particle-induced lung injury.

#### Pulmonary hypertension

Pulmonary hypertension (PH) as a result of narrowed or blocked blood vessels can cause serious damage to the lung arteries, resulting in lung injuries [76]. Due to its complex pathology, there is currently no effective therapeutic strategy to improve PH in lung injuries. However, a recent study suggested that circRNAs may play a protective role during PH development. Through microarray analysis, Wang et al. identified 64 significantly differentially expressed circRNAs in the lung tissues of hypoxia-induced PH mice models. GO and KEGG pathway analyses revealed that circRNA_004592, a miR-152 sponge, is significantly upregulated. As previous studies have shown, miR152 was thought to promote endothelial cell proliferation [77] and regulate glioma cell apoptosis [78]. Hence, the upregulation of circRNA_004592 was suggested to indirectly promote cell proliferation and inhibit cell apoptosis in pulmonary vessels [79]. However, the validity of this study requires further verifications both in vitro and in vivo. Current studies of PH in lung injuries remain preliminary and further studies are required.

#### Acute lung injury

Acute lung injury (ALI) as a result of acute inflammation in the lung can cause severe damages to the vascular endothelium and alveolar epithelium [80]. Current treatments remain ineffective with poor prognosis. Study of circRNAs in ALI suggested that circRNAs may play a role in both aggravating and alleviating ALI.

Han et al. reported a significant upregulation of hsa_circ_0059930 expression in lipopolysaccharide (LPS)-induced ALI MRC-5 cell models. Further analyses suggested that hsa_circ_0059930 acted via the hsa-miR-382-5p/topoisomerase-1 axis. Knockdown of hsa_circ_0059930 showed a significant improvement in cell proliferation and a significant decrease in cell apoptosis during ALI [81]. This suggested that hsa_circ_0059930 may play a critical role in regulating cell proliferation and apoptosis during ALI. Although this study revealed a potential therapeutic prospect of hsa_circ_0059930, the study was only able to perform preliminary screenings of its interactions in vitro. Further studies are recommended to verify the hsa_circ_0059930/hsa-miR-382-5p/topoisomerase 1 interaction through a double luciferase activity assay and RNA immunoprecipitation experiment.

In addition, Zhu et al. reported a novel circRNA, circRNA_0001679, to be significantly upregulated in both ALI-induced mouse lung epithelial (MLE)-12 cells models and septic ALI mouse models. Luciferase reporter assay suggested that circRNA_0001679 positively regulated DUSP16 expression through sponging miR-338-3p. As a result, circRNA_0001679 indirectly induced cell apoptosis and upregulate the expression of proinflammatory cytokines, resulting in the aggravation of ALI [82]. This study provided valuable insight into circRNA_0001679’s role in ALI and provided useful direction for further investigation of its therapeutic prospect in ALI.

Apart from aggravating ALI, studies have also suggested that circRNAs may alleviate ALI both in vitro and in vivo. Li et al. reported that circHECTD1 was significantly downregulated in both ALI mouse and LPS-induced ALI human cell models. Downregulation of circHECTD1 reversed its sponging activity against miR-320a sponge, leading to inhibition of PIK3CA. This attenuated alveolar epithelial cell apoptosis and in turn alleviated ALI [83].

Furthermore, Bao et al. identified 11 significantly upregulated circRNAs and 126 significantly downregulated circRNAs in cecal ligation and puncture-induced acute lung injury mice models [84]. Through GO analysis, the upregulated circRNAs were predicted to be involved in the TGF-β signalling pathway to regulate macrophage development [85]. Further rt-qPCR analysis suggested that the upregulated circRNAs may act via the mmu-miR-7c-5p, mmu-miR-132-3p and miR-124-3p axes to negatively regulate lung macrophages’ M2 polarisation and inhibit anti-inflammatory responses during ALI. On the other hand, the downregulated circRNAs were predicted to mediate histone H3K27 methylation to directly regulate inflammatory response through regulating the expression of crucial transcription factors in macrophage differentiation and polarisation [86].

Overall, current studies suggested that circRNAs may contribute to various critical biological pathways during lung injury. However, most studies focused on discovering novel circRNAs instead of elaborating on the currently identified circRNAs. Further studies are recommended to build on the current studies and to establish a clearer interaction network of the identified circRNAs. Moreover, most reported studies only focused on injury sites within the lung tissues. Prospective studies could investigate the differences between the expression profiles of circRNAs in lung injury patients-derived blood samples and healthy donor-derived blood samples. This would help to identify potential biomarker candidates that may further our understanding of the injury.

### Kidney

#### Mesangial cell injury

Mesangial cells play a critical role in the glomerular function of the kidney. Injury to the mesangial cells may result in various kidney diseases such as diabetic nephropathy [87]. Using a high glucose-induced mesangial cell injury model, the study identified a significant upregulation in circLRP6 expression. CircLRP6 was showed to sponge miR-205 to positively regulate HMGB1. Upregulation of HMGB1 activated the toll-like receptor 4 (TLR4) NF-κB pathway which in turn promoted mesangial cell injury [33]. As this in vitro study was only able to mimic the injury site through high glucose treatment, further studies are recommended to study circLRP6 through in vivo models that better reflect on the microenvironment of mesangial cell injury.

#### Acute kidney injury

Acute kidney injury (AKI) is a sudden decline in kidney functions ranging from minor kidney dysfunction to serious kidney failure [88]. While some may be minor, AKI could lead to severe multi-organ dysfunction. Hence, a promising biomarker in detecting AKI would help to facilitate earlier interventions to prevent the development of AKI-initiated multi-organ dysfunction.

Studies have suggested that circRNAs may play a regulatory role during inflammatory response in AKI. For example, circ_0114428 was identified to be significantly upregulated in sepsis-induced kidney injurous cells. Upregulation of circ_0114428 positively regulated cereblon (CRBN) expression through sponging miR-495-3p [89]. This in turn activated the NF-κB signalling pathway and triggered the release of proinflammatory cytokines including IL-6 and IL-1β [90]. Interestingly, the same upregulation profile of circ_0114428 and CRBN in AKI was also identified in the serum specimens from 33 septic AKI patients. Although the sample size may not be significant, this study revealed an important biomarker candidate and therapeutic target for AKI.

In addition, multiple in vitro studies using LPS-induced AKI human kidney 2 (HK2) cell modes have reported a significantly upregulated expression profiles of numerous circRNAs that may contribute to promoting apoptosis and inflammatory response in AKI. Where circFANCA acted via the miR-93-5p/oxidative stress-responsive kinase 1 (OXSR1) axis [91]; circ_RASGEF1B acted via the miR-146a-5p/pyruvate dehydrogenase kinase 1 (Pdk1) axis [92]; circBNIP3L acted via the miR-370-3p/myeloid differentiation primary response 88 (MYD88) axis [93]; circ_0114427 acted via the miR-495-3p/TNF receptor-associated factor 6 (TRAF6) axis [94]; circSnrk acted via the MAPK axis [27]; hsa_circ_010157 (circSTRN3) acted via the miR-578/TLR4 axis [95]. Apart from in vitro studies, [101]also identified circTLK1 to be significantly upregulated in vivo. With knockdown assay, the study suggested that circTLK1 can positively regulate HMGB1 through sponging miR-106a-5p to mediate oxidative stress, inflammation and apoptosis in sepsis-related AKI [96]. Interestingly, HMGB1 was also identified as a downstream target of circLRP6 mesangial cell injury in kidney [33]. Future studies are recommended to study circRNAs’ direct interacting network with HMGB1 to further understand the therapeutic prospect of circRNAs in kidney injuries.

In a more recent study, Lu et al. reported a significant upregulation in circHIPK3 expression level in septic AKI HK2 cells. CircHIPK3 was suggested to positively regulate FOXA1 through sponging miR-338. This in turn promoted cell apoptosis in AKI [97]. Interestingly, circHIPK3 was also reported to promote cell apoptosis and inflammatory response in spinal cord injury and induce fibroblast activation in idiopathic pulmonary fibrosis. This fundamental deviation of circHIPK3’s roles in different tissue injuries may suggest that circHIPK3 may act in a tissue-specific manner depending on the local cellular environment. However, further research is required to verify this hypothesis to better understand the fundamental mechanism of circHIPK3 in organ injuries.

#### Ischemia/reperfusion kidney injury

Ischemia–reperfusion kidney injury, as a pathological state of acute kidney injury, is a common consequence of major surgery in kidney transplantation [98]. An in vivo study by [101] identified a significant upregulation of circAKT3 expression during renal I/R injury. Upregulated circAKT3 sponged miR-144-5p to activate the Wnt/β-catenin pathway, resulting in the promotion of cell apoptosis [99]. Another in vivo study also reported that circRNAs may promote inflammatory response in kidney I/RI. Circ_001839, a miR-432-3p sponge, was identified as significantly upregulated to positively regulate the expression of inflammatory cytokines (tumour necrosis factor-alpha (TNF-α), interferon-gamma (IFN-γ) and IL-6). This in turn promoted inflammatory response and aggravated renal I/RI [100]. Similarly, circ_0023404 was identified as significantly upregulated in vitro. Circ_0023404 acted via the miR-136/IL-6R axis to promote cell apoptosis and inflammatory response in HK2 cells [101].

While there are both in vitro and in vivo studies of circRNAs in I/R kidney injury, validations are needed to verify the interactions of the identified circRNAs. For example, circ_001839 was reported to positively regulate IL-6 while circ_0023404 was suggested to positively regulate IL-6 receptor, both of which are within the same signalling axis that promote inflammatory response. Future studies are recommended to study the in vivo interactions of multiple circRNA species and their corresponding signalling events in kidney I/RI.

#### Renal fibrosis

Renal fibrosis as a result of kidney injuries leads to progressive loss of kidney function in chronic kidney disease [102]. It is important to identify a biomarker that can give successive estimations of the degree of renal fibrosis during chronic kidney disease. This will help clinicians to better access the prognosis and design more effective treatment plans for the patients [103].

A recent study by Zhou et al. examined chronic kidney disease patients-derived renal tissues. CircPlekha7, a miR-493-3p sponge, was identified as significantly downregulated. This led to the inhibition of Kruppel-like factor 4 (KLF4) expression [104]. As KL4 was previously shown to inhibit inflammatory and fibrotic responses in proximal tubule cells [105], the study proposed that the downregulation of circPlekha7 and KLF4 may be a critical factor that contributed to the aggravation of renal fibrogenesis. As the identified circRNA in this study was derived directly from patients with chronic kidney disease, its therapeutic prospects seem promising. Further studies are recommended to verify this result in vivo.

#### Lupus nephritis

As an autoimmune disease, lupus nephritis can lead to severe inflammation in the kidney. Luan et al. examined six renal biopsies from lupus nephritis class IV patients and identified 171 significantly differentially expressed circRNAs with 142 circRNAs upregulated and 29 circRNAs downregulated. In particular, further analyses of an upregulated species—circHLA-C—suggested that it may act via the miR-150 axis to participate in the pathogenesis of lupus nephritis [106]. However, the study were preliminary and further in vitro and in vivo studies are needed to verify circHLA-C’s interaction with miR-150 in lupus nephritis.

### Heart

Our previous study has identified that m6A modification on long non-coding RNAs may play a regulatory role during the regeneration and development of mouse myocardium [107]. In particular, recent studies have also identified multiple circRNAs to play a regulatory in heart injuries.

#### Myocardial ischemia/reperfusion injury

Common surgical treatment for acute myocardial infarction usually leads to myocardial ischemia/reperfusion injury [108]. Studies on circRNAs in myocardial I/RI suggested that circRNAs may promote apoptosis and inflammation in aggravating myocardial I/RI. An i*n vitro* study by Zhang et al. identified circ_0030235 to be significantly upregulated in OGD/R-induced H9C2 cell models. Further analysis suggested that circ_0030235 may act via the miR-526b axis to inhibit the PI3K/Akt and MAPK pathways. As a result, inhibition of the PI3K/Akt pathway led to apoptosis and hence, aggravated myocardial I/RI [30]. Furthermore, a study by Wang et al. also suggested a similar biological function of circRNAs in myocardial I/RI. Through hypoxia/reoxygenation (H/R) H9C2 cell models, the study identified circ_0001206 to be significantly downregulated. Circ_0001206 was suggested to sponge miR-665 to regulate the expression of Crk-like protein (CRKL). Hence, downregulation of circ_0001206 led to CRKL inhibition that in turn promoted cardiomyocyte apoptosis [109]. While these studies were able to successfully demonstrate the critical roles circRNAs played in myocardial I/RI, the underlying molecular interactions remain vague and further studies are required to investigate in the precise mechanisms of the reported circRNAs.

Apart from in vitro studies, in vivo studies of circRNAs in myocardial I/RI also suggested that circRNAs may play a role in regulating cardiomyocyte apoptosis. For example, Hu et al. identified significant upregulation of circSAMD4A in the heart tissues of acute myocardial infarction (MI)-mice models and H/R induced H9C2 cells. Further analysis suggested that circSAMD4A may act via the miR-138-5p axis to positively regulate the expression of inflammatory cytokines (IL-1β, TNF-α and IL-6) and negatively regulate anti-apoptotic factors (BCL2 and Bax) [110]. Hence, the study suggested that upregulation of circSAMD4A may aggravate H/R-induced apoptosis and inflammation. However, further studies are recommended to explore the detailed mechanism of the circSAMD4a/miR-138-5p axis and its other potential downstream effector proteins.

In addition, Sun et al. identified significant downregulation in circFOXO3 expressions in MI-induced myocardial injured rats. As previously discussed, circFOXO3 was also reported to maintain BBB integrity in brain I/RI and to promote inflammatory responses in cigarette particle-induced lung injury. In contrast to the two injuries, circFOXO3 was suggested to inhibit inflammatory response in myocardial injury. Through overexpression of circFOXO3, the study identified a significant downregulation in lysine acetyltransferase 7 (KAT7) expression which in turn alleviated OGD-Induced cardiomyocyte injury in vitro [111]. As circFOXO3 was reported to exhibit different biological functions across all three types of organ injuries, further studies are required to explore the differences in the underlying mechanisms of circFOXO3 in each organ in vivo to help verify the therapeutic potential of circFOXO3 in organ injuries.

Moreover, with both in vivo and in vitro models, Liu et al. reported that circRbms1 was significantly upregulated in the heart tissues of MI mice and hypoxia-induced cardiomyocyte cell models. Further analysis suggested that circRbms1 acted via the miR-742-3p/FOXO1 axis to aggravate hypoxia-induced cardiomyocyte injury [112]. Similarly, Wang et al. identified a significantly downregulated circRNA, circUBXN7, in acute MI mice and H/R treated cells. CircUBXN7 was found to exhibit miR-622 sponging activity to positively regulate MCL1 which served to suppress cell apoptosis and inflammatory responses. As a result, the downregulation of circUBXN7 during I/RI reversed this inhibitory effect and resulted in promoting cell apoptosis and inflammatory responses [113]. While both studies were able to verify the identified circRNAs’ roles in promoting cell apoptosis and inflammatory responses in hypoxia-induced cardiomyocyte injury, the exact molecular mechanisms remain unclear. To further investigate the therapeutic prospect of the identified circRNAs, it is important to characterise the molecular mechanisms of these circRNAs more precisely.

Apart from circRNAs’ role in regulating apoptotic and inflammatory responses, studies have also identified circRNAs as potential therapeutic targets in myocardial I/RI. For example, Cheng et al. identified circPostn to be significantly upregulated in myocardial infraction patient plasma, myocardial infraction mouse models and H/R treated human cardiomyocytes. Further analyses suggested that circPostn acted via the miR-96-5p/BCL2 interacting protein 3 (BNIP3) axis to positively regulate BNIP3 and promote apoptosis. As a BCL2 interacting protein, BNIP3 attenuated anti-apoptotic protein BCL2 expression and promoted pro-apoptotic protein Bax and cleaved caspase-3 expression. Importantly, a previous study has established that targeting BNIP3 in inflammation-related cardiac failure has a promising therapeutic potential [114]. Hence, the study proposed that the circPostn/miR-96-5p/BCL2 axis may be a potential therapeutic target for MI-induced myocardial injury [115].

Lastly, studies have also identified circRNA as a potential biomarker in myocardial I/RI. Bian et al. identified a significantly upregulated circRNA, circHelz, in the ischemic myocardium of MI mouse models. CircHelz was suggested to positively regulate NLRP3 through sponging miR-133a-3p. As a result, upregulation of NLRP3 activated the NLRP3 inflammasome pathway, leading to inflammatory responses that aggravated myocardial injury in mouse ischemic hearts [116]. While the study was able to identify the molecular mechanism of circHelz-induced inflammatory response, future studies are recommended to examine patient-derived samples to verify the biomarker potential of circHelz in clinical settings.

#### Cardiac fibrosis

Cardiac fibrosis as a result of imbalanced extracellular matrix (ECM) production and degradation can lead to severely impaired heart muscle function [117]. The pathology of cardiac fibrosis mainly involves robust fibroblast proliferation and fibroblast-to-myofibroblast differentiation. Studies of circRNAs in cardiac fibrosis have suggested that circRNAs may play a role in both biological processes. Zhu et al. found a significant downregulation of circNFIB (mmu_circ_0011794) expression in mice post-MI heart samples and TGF-β-treated cardiac fibroblast cell models. CircNFIB, as a miR-433 sponge, was found to positively regulate antizyme inhibitor 1 (AZIN1) to activate the TGF-β–Smad3 signalling pathway [118]. Further knockdown assay of AZIN1 confirmed that downregulation of AZIN1 promoted fibroblast proliferation and cardiac fibroblast-to-myofibroblasts differentiation [119]. Hence the study suggested that the downregulation of circNFIB may contribute to cardiac fibroblast proliferation and differentiation during cardiac fibrogenesis.

In addition, studies have also suggested that circRNAs may contribute to the development of fibrotic phenotype during cardiac fibrosis. Tang et al. identified significant upregulation of circRNA_000203 expression in diabetic mouse myocardium and angiotensin-II-induced mouse cardiac fibroblasts. As a miR-26b-5p sponge, circRNA_000203 was suggested to positively regulate alpha-smooth muscle actin (α-SMA), collagen1a2 (Col1a2) and connective tissue growth factor (CTGF) in cardiac fibroblasts [120]. α-SMA, Col1a2 and CTGF are all fibrosis-associated proteins that exhibit pro-fibrosis effects during cardiac fibrosis. Hence, the study suggested that circRNA_000203 may play a critical role in regulating the development of fibrotic phenotype in cardiac fibrogenesis. Moreover, Ni et al. also identified another circRNA, circHIPK3, to play a similar role in cardiac fibrosis. Through PCR and Sanger sequencing technique, the study reported a significant upregulation in circHIPK3 expression level in angiotensin-II-induced mouse cardiac fibroblasts. Further analysis suggested that circHIPK3 may act via the miR-29b-3p axis to positively regulate α-SMA, Col1a1 and Col3a1 expression, hence contributing to cardiac fibrogenesis [121]. Interestingly, circHIPK3 was also reported in spinal cord injury, idiopathic pulmonary fibrosis and acute kidney injury. While circHIPK3’s functions in SCI and AKI were different, circHIPK3 in IPF was suggested to play a similar role in promoting fibrogenesis during pulmonary fibrosis. In IPF, circHIPK3 was suggested to promote fibroblast proliferation and fibroblast-to-myofibroblast transition in the lung. Whereas in cardiac fibrosis, circHIPK3 was suggested to regulate ECM content such as promoting α-SMA and collagens expression. Although circHIPK3 acted via two different axes in these two injuries, the similar pathology outcome may suggest that circHIPK3 could function in an environment-dependent manner (i.e., the same circRNA species exhibit similar biology function in the pathology of general organ fibrosis). However, this is only a speculation, and more studies are needed to verify this fundamental molecular nature of circHIPK3.

### Liver

#### Hepatic fibrosis

Hepatic fibrosis as a result of repeating wound healing processes during kidney injuries could lead to various degrees of liver failure if treated inappositely [122]. To improve the prognosis for hepatic fibrosis, effective biomarkers that can detect early-stage hepatic fibrosis are needed. Three significantly downregulated circRNAs—mmu_circ_0001682, mm9_circ_006613 and circPSD3—were identified to inhibit hepatic fibrosis activation in CCl4-induced hepatic fibrosis mice. Further analysis of circPSD3 suggested that it may act via the miR-92b-3p/Smad7 axis to regulate the expression of Smad7 [123]. As previous studies have reported, Smad7 negatively regulate the TGF-β1/Smad signalling pathway in hepatic fibrosis [124]. Therefore, as a result of circPSD3 downregulation, Smad7 is subsequently downregulated, leading to activation of TGF-β1/Smad signalling pathway. This led to the release of pro-inflammatory cytokines and the suppression of hepatic stellate cells (HSCs) activation and proliferation. The therapeutic prospects of these circRNAs still require further verification as the study was only able to correlate the identified circRNA’s functions through GO and KEGG analyses matches. While this may reflect on circRNAs’ roles to a certain extent, it failed to recognise other potential interactions and regulatory functions that these circRNAs may exhibit.

Furthermore, study has also suggested that circRNAs may play a vital role in the signal transduction pathway during hepatic fibrosis. Using the HSCs model, Zhu et al. study identified a significant upregulation in circ_0067835 expression. Circ_0067835, as a miR-155 sponge, was found to positively regulate the Akt/FOXO3a signalling pathway [125]. As a previous study has reported, the activation of Akt/FOXO3a signalling pathway was associated with HSCs proliferation [126]. Hence, circ_0067835 was suggested to indirectly regulate HSC proliferation in hepatic fibrosis.

#### Hepatic ischemia/reperfusion injury

Hepatic ischemia/reperfusion injury in post-liver surgery or transplant is a common cause of liver dysfunction and/or failure. It involves ischemia-mediated cellular damage and may lead to multi-organ dysfunction syndrome (MODS) or systemic inflammatory response syndrome (SIRS) [127]. The current effective intervention of I/RI uses ischemic preconditioning (IPC), where a short period of ischemic/reperfusion is induced through brief vascular occlusion [128]. Importantly, a recent study of circRNAs in hepatic I/RI has found that circRNAs may play a role during IPC in hepatic I/RI.

Using hepatic artery occlusion-induced mice models, 43 significantly upregulated circRNAs and 7 significantly downregulated circRNAs were found in IPC-treated ischemic mice liver cells. Further analyses identified that circRNA_017753 exhibited sponging activities against miR-218-5p, miR-7002-3p and miR-7008-3p to regulate the Jade Family PHD Finger 1 (Jade1) signalling pathways [129]. Jade1, as a component of the HBO1 complex, was reported as a key regulator of cell cycle progression, redifferentiation and apoptosis [130]. Therefore, circRNA_017753 may be a critical regulator in the protection mechanism of IPC during hepatic I/RI. However, the exact causation relationship and molecular mechanisms were not well explored. Further studies are recommended to investigate in the underlying molecular mechanisms to better understand circRNAs’ roles in IPC.

#### Radiation-induced liver injury

Radiation-induced liver fibrosis (RILF) is a common complication of radiation therapy for liver cancer. Using irradiated HSCs, Chen et al. identified 809 significantly differentially expressed circRNAs with 179 circRNAs upregulated and 630 downregulated. Further GO and KEGG pathway analyses suggested that a significantly upregulated circRNA, hsa_circ_0071410, may act via the miR-9-5p axis [131]. As previous studies have reported, miR-9-5p may inhibit the activation and proliferation of HSC by targeting multidrug resistance‑associated protein 1 (MRP1) [132]. Hence, by sponging miR-9-5p, hsa_circ_0071410 was suggested to attenuate hepatic fibrosis activation and alleviate RILF.

Furthermore, Niu et al. identified a significantly upregulated circRNA, circTUBD1, in irradiation and LPS-induced HSCs. CircTUBD1, as a miR-146a-5p sponge, can positively regulate the expression of TLR4, interleukin 1 receptor associated Kinase 1 (IRAK1) and TRAF6, leading to the activation of the TLR4/NF-κB signalling pathway [133]. This in turn promoted the production of pro-inflammatory cytokines and triggered inflammatory response in irradiation-induced liver injury. Similarly, also using irradiation and LPS-induced HSC models, Chen et identified another significantly upregulated circRNA, circRSF1, to promote inflammatory response during irradiated-induced hepatic injury. Using luciferase reporter assay, the study found that circRSF1 may act via the miR-146a-5p/ras-related C3 botulinum toxin substrate 1 (RAC1) axis. As previous studies reported, RAC1, a member of the Rho family of GTPases, is an intracellular transduce that can activate the Sonic Hedgehog pathway to promote HSCs activation [134] and can act on NF-κB and c-Jun N-terminal kinases (JNK) to promote inflammatory responses in HSCs [135]. Hence, the upregulation of circRSF1 led to the upregulation of RAC1 which promoted proinflammatory responses in irradiation-induced liver injury [136]. Overall, current studies of circRNAs in irradiation-induced liver injury were mostly conducted in vitro with human satellite cells. Future studies are recommended to verify the identified circRNAs and their roles in vivo.

### Intestinal injury

Intestinal injury as a result of trauma can lead to various extents of severity depending on the types of injuries. Students have suggested that circRNAs may play a regulatory in the repair process of intestinal injury. Recent study by Zhang et al. identified 308 significantly dysregulated circRNAs in the intestinal mucosal cell of severely burnt mice. Of the dysregulated circRNAs, 97 circRNAs were identified as upregulated and 211 circRNAs were identified as downregulated [137]. In particular, further analyses of significantly downregulated circRNAs suggested that circRNA_Maml2 can positively regulate FZD7 through sponging miR-93-3p. As a G-protein coupled receptor protein, FZD7 can bind to Wnt ligands to activate both canonical and non-canonical Wnt signalling pathways, leading to cell proliferation, differentiation and migration [138]. Hence, the study suggested that circRNA_Maml2 may play a vital role in the repair of intestinal mucosa through the miR-93-3p/FZD7/Wnt/β-catenin pathway [28].

Similarly, this role of circRNAs was further demonstrated in the study by Xiao et al. Through in vivo mouse models, the study identified a significant downregulation in the expression of circHIPK3 during polymicrobial sepsis-induced intestinal epithelial injury. Further analyses suggested that circHIPK3 acted via the miR-29b axis to regulate the expression of RAC1, Cdc42 and cyclin B1. Hence, circHIPK3 downregulation attenuated RAC1, Cdc42 and cyclin B1 expressions. As key cell-cycle regulators, the downregulation of RAC1, Cdc42 and cyclin B1 led to the inhibition of cell proliferation, which in turn aggravated intestinal injury. In confirmation of this mechanism, the study showed that overexpression of circHIPK3 can reverse the attenuation of cell proliferation and increase intestinal epithelial cell proliferation in mice [139]. Interestingly, as previously discussed, circHIPK3 was also identified in spinal cord injury, idiopathic pulmonary fibrosis, acute kidney injury and cardiac fibrosis. Of which, circHIPK3 was reported to exhibit similar biological functions between SCI and AKI and between IPF and cardiac fibrosis. While previous studies in other organ injuries have not yet suggested that circHIPK3 can regulate key cell-cycle regulators, its role in intestinal injury was not tissue-specific. As previous studies have reported, circHIPK3 was found to be abundantly expressed across various cell types and has been associated with cell growth and proliferation [140]. Therefore, future studies are recommended to further explore circHIPK3’s function in regulating the cell cycle in other tissue injuries and compare the underlying differences in its molecular mechanisms.

### Reproductive system: uterus and ovary injury

Uterus and ovary injury as a result of traumatic rupture to the uterus or ovary can lead to various extents of damage depending on the significance of the injury. Recent studies suggested that circRNAs may play a role during the damaging process of uterus and ovary injury. Using in vivo mouse models, Li et al. reported a significant upregulation in circScar expression and a significant downregulation in circZC3H4 expression upon procymidone dose-dependent uterus and ovary damage [141]. The former, circScar, has been previously reported to quench ATP synthase β-subunit (ATP5B) [142], leading to inhibition of ATP production and subsequent ovarian cell proliferation [143]. The latter, circZC3H4, can positively regulate ZC3H4 through sponging miR-212 [144]. Hence, the downregulation of circZC3H4 led to the upregulation of miR-212 which subsequently inhibited ZC3H4 expression. As an RBP, downregulation of ZC3H4 resulted in higher expression of JNK1, JNK2 and JNK3 which led to follicle damage and cell apoptosis, thus aggravating uterine damage [145]. Interestingly, circZC3H4 was also reported in silicosis lung injury to play a role in fibroblast activation and proliferation. While its role in lung injury differs from the reported role in uterus and ovary injury, their molecular mechanisms of targeting ZC3H4 protein may be identical. Hence, further studies are required to verify circZC3H4’s roles in other organ injuries to better understand its therapeutic prospect.

## Future prospects of circRNAs

Although current studies have highlighted the critical roles circRNAs play in organ injuries, their therapeutic prospects remain inconclusive. CircRNAs have been shown to act via several important biological pathways—cell proliferation (MAPK pathway), apoptosis (PI3K/Akt pathway) and inflammation (NF-κB pathway)—across different types of organ injuries. However, due to the complex intracellular signalling networks, most studies were only able to identify circRNA’s interaction within a single mechanism axis. With the ability to act as miRNA sponges, protein decoys and transcription regulators, circRNAs may exhibit alternative biological functions that we have not yet fully explored and understood. Hence, without a better understanding of the underlying intracellular interactions, the exact therapeutic potential of circRNAs remains unclear. In addition, most studies of circRNAs in organ injuries did not present a direct causation relationship between an identified circRNA species and a specific pathology outcome. Some studies were only able to demonstrate bioinformatics analyses of the potential downstream targets of circRNAs and correlate the identified targets with their respective roles in a particular injury. While the others were only able to show a direct outcome through an overexpression or knockdown system. Either method only provided a limited understanding of the molecular mechanism of circRNAs in vivo. Moreover, the applicability of circRNAs as therapeutic targets requires further investigation. Although an overexpression or a knockdown system is achievable in a laboratory environment, it is not applicable to directly translate such systems in clinical settings. Therefore, future studies should explore alternative delivery systems that can be applied appropriately in clinical settings.

However, with these in mind, the potential therapeutic prospects of circRNAs cannot be ruled out. Firstly, circRNAs’ covalently enclosed-loop structure gives them a relatively long half-life and stability in both nucleus and cytoplasm [146]. Thus, dysregulation in circRNA’s expression profiles can be readily detected. Secondly, circRNAs were found highly abundant and exhibited significantly dysregulated expression profiles across all types of organ injuries from CNS to major visceral organs such as the lung, kidney and heart. Thirdly, studies of circRNAs in organ injuries with similar pathology have shown that circRNAs may act in a pathology-dependent manner. For example, in organ fibroses, circRNAs have been suggested to serve the same function of activating fibroblast and promoting cell proliferation across different organs [147].

While directly applying circRNAs as therapeutic targets may still be challenging, utilising circRNAs as disease and injury biomarkers may be relatively achievable. As discussed, circRNAs were found to regulate key cellular functions including cell proliferation and inflammation. The activation level of these pathways can therefore indicate a certain extent of the degree and severity of the diseases and injuries [148]. Hence, as a direct regulator, changes in the expression level of circRNAs may directly reflect on the disease pathology. Future research should identify a more defined and specific circRNA marker in each organ injury and aims to develop an efficient detection system of circRNAs that can be applied to a clinical setting.

## Conclusion

Current studies suggested that circRNAs may be a key player in regulating cell proliferation, cell apoptosis and inflammatory response during major organ injuries. Through comparing studies from different organ injuries with similar pathology, we suggested that circRNAs function in both tissue-specific and pathology-specific manner (e.g., circRNAs promote fibroblast activation in organ fibrosis). However, there were a few limitations in the current studies. Firstly, most of the current studies were conducted on in vitro human cell models and in vivo animal models. There were only a few studies that examined patient-derived samples. Secondly, current studies were only able to explore the function of individual circRNA. It is important to recognise that the intracellular environment is extremely dynamic. Further studies are recommended to study the interactions between multiple circRNAs species in a microenvironment that can more closely resemble the intracellular condition of a specific disease pathology during organ injury. Lastly, with the increasing attention to this research hotspot, more and more circRNAs are being reported and recorded into large databases such as circBase.org. Therefore, to appropriately measure the true applicability of circRNAs in clinical settings, future studies should establish a standardised protocol in this area of research [149].

## Data Availability

The data supporting the conclusions of this article are included within the article and are available in the reference section.

## Abbreviations

AKI: Acute Lung Injury
APC: Adenomatous polyposis coli
AMPK: Adenosine 5′ monophosphate-activated protein kinase
α-SMA: Alpha-smooth muscle actin
AZIN1: Antizyme inhibitor 1
ATP5B: ATP synthase β-subunit
BCL2: B-cell lymphoma-2
BAD: Bcl-2-associated death promoter
Bax: Bcl-2-associated X protein
BNIP3: BCL2 interacting protein 3
BBB: Blood–brain barrier
CK1: Casein kinase 1
CDK2: Cell division protein kinase 2
CNS: Central nervous system
CRBN: Cereblon
CDR1as: Cerebellar degeneration-related 1 antisense
CXCL12: Chemokine (C-X-C motif) ligand 12
COPD: Chronic obstructive pulmonary disease
CircRNA: Circular RNAs
CiRNAs: Circular intronic RNAs
Col1a2: Collagen1a2
CeRNAs: Competing endogenous RNAs
CTGF: Connective tissue growth factor
CRKL: Crk-like protein
P21: Cyclin-dependent kinase inhibitor 1
JNK: C-Jun N-terminal kinases
DEX: Dexmedetomidine
Dram2: DNA damage regulated autophagy modulator 2
DUSP1: Dual specificity phosphatase 1
EcircRNAs: Exonic circular RNAs
EIciRNAs: Exon–intron circular RNAs
ECM: Extracellular matrix
FZD: Frizzled
Lgals3: Galectin 3
GO: Gene Ontology
GSK3: Glycogen synthase kinase 3
GDF11: Growth differentiation factor 11
Grb2: Growth factor receptor-bound protein 2
GEF: Guanine nucleotide exchange factor
HSC: Hepatic stellate cell
HMGB1: High mobility group box 1
HK2 cell modes: Human kidney 2 cell models
H/R: Hypoxia/reoxygenation
IPF: Idiopathic pulmonary fibrosis
IKK-B: Inhibitor of nuclear factor kappa-B-kinase
IFN-γ: Interferon-gamma
IRAK1: Interleukin 1 receptor associated Kinase 1
IL-6: Interleukin-6
HT22 cell model: Immortalised mouse hippocampal cell model
IPC: Ischemic preconditioning
I/RI: Ischemia/reperfusion injury
Jade1: Jade Family PHD Finger 1
KLF4: Kruppel-like factor 4
KEGG: Kyoto Encyclopedia of Genes and Genomes
LPS: Lipopolysaccharide
LRP5/6: Low-density lipoprotein receptor-related proteins 5 and 6
LEF: Lymphoid enhancer-binding factor
KAT7: Lysine acetyltransferase 7
MRNAs: Messenger RNAs
MiRNA: MicroRNA
MREs: miRNA response elements
MAPK: Mitogen-activated protein kinase
MLE: Mouse lung epithelial
MRP1: Multidrug resistance‑associated protein 1
MODS: Multi-organ dysfunction syndrome
MYD88: Myeloid differentiation primary response 88
MI: Myocardial infraction
NLRP3: NOD-, LRR- and pyrin domain-containing protein 3
NF-κB: Nuclear factor kappa-light-chain-enhancer of activated B cells
OXSR1: Oxidative stress-responsive kinase 1
OGD/R: Oxygen–glucose deprivation and reperfusion
PPRs: Pattern-recognition receptors
PIP2: Phosphatidylinositol (3,4)-bisphosphate
PIP3: Phosphatidylinositol (3,4,5)-trisphosphate
PI3K: Phosphoinositide-3-kinase
Akt: Protein kinase B
PH: Pulmonary hypertension
Pdk1: Pyruvate dehydrogenase kinase 1
QKI: Quaking
OGD/R: Oxygen and glucose deprivation/reoxygenation
RILF: Radiation-induced liver fibrosis
RAC1: Ras-related C3 botulinum toxin substrate 1
ROS: Reactive oxygen species
RTK: Receptor tyrosine kinases
Rt-qPCR: Reverse transcription-quantitative polymerase chain reaction
RBPs: RNA-binding proteins
SRB1: Scavenger Receptor Class B Type 1
SCI: Spinal cord injury
SOS: Sone of sevenless
SIRS: Systemic inflammatory response syndrome
TXNIP: Thioredoxin-interacting protein
TRAF6: TNF receptor-associated factor 6
TLR-4: Toll-like receptor 4
TGF-β: Transforming growth factor-beta
TMCAO: Transient middle cerebral artery occlusion
TBI: Traumatic brain injury
TNF-α: Tumour necrosis factor-alpha
TCF: T-cell factor
ZC3H4: Zinc finger CCCH-type containing 4
ZO-1: Zonula occludens-1
